# Thymoma negatively affects the neurological outcome of myasthenia gravis after thymectomy: a propensity score matching study

**DOI:** 10.1186/s13019-024-02511-6

**Published:** 2024-01-31

**Authors:** Wenxin Tian, Hanbo Yu, Yaoguang Sun, Jing He, Qingjun Wu, Chao Ma, Peng Jiao, Chuan Huang, Donghang Li, Hongfeng Tong

**Affiliations:** 1 Department of Thoracic Surgery, Beijing Hospital, National Center of Gerontology, Institute of Geriatric Medicine, Chinese Academy of Medical Sciences, No.1 Dahua Road, Dong Dan, Beijing, 100730 People’s Republic of China; 2 Department of Neurology, Beijing Hospital, National Center of Gerontology, Institute of Geriatric Medicine, Chinese Academy of Medical Sciences, Beijing, People’s Republic of China

**Keywords:** Thymoma, Myasthenia gravis, Neurological outcome, Thymectomy, Propensity score matching

## Abstract

**Background:**

Thymoma and myasthenia gravis (MG) interact with each other. This study aimed to evaluate the effects of thymoma on neurological outcome of MG patients after thymectomy using the propensity score matching (PSM) method.

**Methods:**

Consecutive patients with MG who underwent thymectomy at Beijing Hospital between January 2012 and August 2021 were retrospectively enrolled. Clinical and follow-up data were collected. Statistical analysis was performed using SPSS 23.0 software. PSM was performed to eliminate selection bias.

**Results:**

A total of 456 patients were included in this study. Thymoma was present in 138 (30.3%) patients. The median follow-up time was 72 (range, 12–135) months. At the last follow-up, a lower proportion of thymomatous MG patients achieved complete stable remission (CSR) compared with non-thymomatous MG patients (*P* = 0.011), and the effective rate [CSR + pharmatologic remission (PR) + minimal manifestations (MM)] of thymomatous MG patients was also lower (*P* = 0.037). Considering time to CSR, Kaplan–Meier analysis showed thymomatous MG patients had lower cumulative CSR rate than non-thymomatous MG patients (log-rank, *P* = 0.019). After PSM, 105 pairs of patients were matched successfully. For the matched patients, thymomatous MG patients had a lower CSR rate and a lower effective rate (*P* = 0.002, 0.039, respectively), and K–M analysis still showed thymomatous MG patients had lower cumulative CSR rate (log-rank, *P* = 0.048). Multivariate Cox analysis demonstrated that thymoma (HR: 0.592, 95% CI 0.389–0.900, *P* = 0.014), older age at the time of surgery (HR: 0.971, 95% CI 0.953–0.990, *P* = 0.003), and preoperative course of MG > 12 months (HR: 0.474, 95% CI 0.317–0.708, *P* = 0.000) were negative predictive factors for CSR.

**Conclusions:**

Thymoma had a negative effect on the neurological outcome of MG after thymectomy. MG patients with old age and a preoperative course of longer than one year had a lower probability of achieving CSR.

## Introduction

Myasthenia gravis (MG) is an autoimmune disease, affecting the neuro-muscular junction, which is characterized by weakness of the skeletal muscles mediated by auto-antibodies, including acetylcholine receptor (AchR) antibodies, muscle-specific tyrosine kinase (MuSK) antibodies, etc. [1]. The pathogenesis of MG is associated with thymic abnormalities, mainly including lymphofollicular hyperplasia and thymoma, and the removal of thymus is considered as an effective treatment to manage MG [2–5]. However, the pathogenesis of MG is different between MG patients with and without thymoma [6], which may result in different responses to thymectomy. Some scholars pointed out that MG patients with thymoma were less likely to attain a complete stable remission (CSR) after thymectomy compared with MG patients without thymoma [7–10], while other studies showed that thymoma did not significantly affect the neurological outcome of MG [11–13]. The present study aimed to evaluate whether thymoma could affect the outcome of thymectomy to manage MG using the propensity score matching (PSM) method in a large MG cohort in China.

## Patients and methods

Consecutive MG patients who underwent thymectomy in Beijing Hospital (Beijing, China) from January 2012 to August 2021 were included in this study. Patients with incomplete medical records or with a postoperative follow-up period shorter than 1 year were excluded. This study was approved by the Institutional Review Board of Beijing Hospital (Approval No. 2021BJYYEC-169-02). Informed consent was waived because no additional treatment was planned (retrospective study).

Clinical data were collected, including gender, age at the time of surgery, onset age of MG, preoperative course of MG, preoperative Osserman classification, serum antibody status, dosage of pyridostigmine bromide, surgical procedure, surgical duration, perioperative myasthenic crisis, thymic pathology, World Health Organization (WHO) type and Masaoka-Koga stage of thymomas, etc. The diagnosis of MG was confirmed by clinical presentations and the following specific tests: neostigmine test, testing of auto-antibodies (AchR, MuSK, etc.), and neuroelectrophysiological tests. Preoperative MG clinical severity was graded by the Osserman classification system [14]. The diagnosis of MG was assessed by a skilled neurologist.

Patients were recommended to continue outpatient or inpatient treatment in the Neurology Department after discharge from the Department of Thoracic surgery. The therapeutic strategies for MG after thymectomy were not standard, which were affected by several factors, including the general conditions of patients, the severity of MG, pathology of thymic lesions, and a physician's preference. Postoperative radiotherapy was suggested to thymomatous patients with Masaoka stage II–IV or incompletely resected tumors (R1/R2 resection). Postoperative chemotherapy was suggested to patients with Masaoka stage IV or R2 resected tumors. All patients were followed up every 6 months in the first 2 years after surgery, and annually thereafter. Follow-up data were collected, including survival status, medications for MG, treatment effect on MG, and thymoma recurrence after surgery. The postoperative effect on MG was examined by a skilled neurologist according to the Myasthenia Gravis Foundation of America criteria defining the post-intervention status [15]. Complete stable remission (CSR) is defined that the patient has had no symptoms or signs of MG for at least 1 year and has received no therapy for MG during that time. Pharmacologic remission (PR) is defined using the same criteria as for CSR except that the patient continues to take some form of therapy for MG, while patients taking cholinesterase inhibitors are excluded. Minimal manifestations (MM) is defined that the patient has no symptoms of functional limitations from MG, but has some weakness on examination of some muscles. Follow-up period is defined as the interval between the date of surgical resection and death or last follow-up. The length of achieving remission is defined as the interval between the date of surgical resection and the date of achieving CSR. The crude CSR rate is defined as number of patients achieving CSR at the last follow-up divided by number of MG patients undergoing thymectomy. The effective rate is defined as number of patients achieving CSR, PR, and MM divided by number of MG patients undergoing thymectomy.

### Statistical analysis

Patients were categorized into two cohorts, including patients with thymoma and those without. For continuous variables, normality test was carried out. Continuous variables with normal distribution were compared using Student’s *t*-test, continuous variables with abnormal distribution were compared using Mann–Whitney U test, and categorical variables were compared using Pearson’s Chi-square test. Survival curves and cumulative CSR curves were generated by the Kaplan–Meier (K–M) method, and the log-rank test was applied for making comparison. Univariate and multivariate Cox proportional hazard models were used to analyze the statistical significance of associations of clinical variables with CSR.

PSM was performed for patients with and without thymoma using seven variables, including age at the time of surgery, gender (male/female), onset age of MG (< 50 years/ ≥ 50 years), preoperative course of MG (≤ 12 months/ > 12 months), surgical procedure (VATS/sternotomy), Osserman classification, and dosage of pyridostigmine bromide. Patients were matched 1:1 using the nearest neighbor matching with a caliper distance of 0.2. In matched groups, paired t-test was utilized to assess continuous variables, and categorical variables were analyzed by the McNemar’s test.

Statistical analysis was performed using SPSS 23.0 software (IBM, Armonk, NY, USA). A two-sided *P* < 0.05 was considered statistically significant.

## Results

### Demographic data

A total of 535 patients with MG underwent thymectomy at Department of Thoracic Surgery of Beijing Hospital from January 2012 to August 2021. After excluding patients with incomplete records or with a postoperative follow-up shorter than one year, 456 MG patients were finally included in this study, of whom 234 patients were men, and 222 were women. The mean age at the time of surgery was 47.1 ± 15.7 years. The median onset age of MG was 47 (range, 1–86) years. The median preoperative course of MG was 6 (range, 0.13–480) months. According to the Osserman classification system, there were 99 (21.7%) patients with type I, 126 (27.6%) patients with type IIa, 195 (42.8%) patients with type IIb, 19 (4.2%) patients with type III, and 17 (3.7%) patients with type IV. 197 (43.2%) patients had information on antibody test. Approximately, 97.5% (192/197) of patients were AChR-positive, none of those patients were MUSK-positive, and 2.5% (5/197) were double-seronegative. 49 (10.7%) patients suffered from myasthenic crisis within 1 month after thymectomy. Thymoma was present in 138 (30.3%) patients, while thymic hyperplasia was present in 280 (61.4%) patients, and thymic atrophy was present in 38 (8.3%) patients. For patients with thymoma, 134 (97.1%) patients received radical resection, and 4 (2.9%) patients were R1-resection.

The sex ratio (male/female) was comparable between thymomatous MG patients and non-thymomatous MG patients (66/72 vs. 168/150, *P* = 0.326). Thymomatous MG patients were older at the time of surgery (50.0 ± 14.0 vs. 45.9 ± 16.3 years, *P* = 0.007), and a higher proportion of thymomatous MG patients with a preoperative course of MG ≤ 12 months was found (115/138 vs. 189/318, *P* = 0.000). More non-thymomatous MG patients had mild symptoms (Osserman I + IIa) (168/318 vs. 57/138, *P* = 0.024). The results of comparison of clinical data between thymomatous MG patients and non-thymomatous MG patients are presented in Table 1.

**Table 1:** Comparison of clinical data between thymomatous MG patients and non-thymomatous MG patients

Variables	Non-thymomatous MG patients	Thymomatous MG patients	T-test or x^2^	*P* value
N	318	138		
Age (years), mean ± SD	45.9 ± 16.3	50.0 ± 14.0	− 2.717	0.007
Sex (male/female)	168/150	66/72	0.965	0.326
Onset age of MG (< 50 years/ ≥ 50 years)	181/137	68/70	2.268	0.132
Preoperative course of MG (≤ 12 months/ > 12 months)	189/129	115/23	24.736	0.000
Dosage of pyridostigmine bromide(mg/d)	180 (0,540)	180 (0,600)	− 1.678	0.095
Osserman classification, n (%)		10.811	0.029	
I	74 (23.3%)	25 (18.1%)		
IIa	94 (29.6%)	32 (23.2%)		
IIb	132 (41.5%)	63 (45.7%)		
III	8 (2.5%)	11 (8.0%)		
IV	10 (3.1%)	7 (5.1%)		
Surgical procedure (VATS/Sternotomy)	298/20	91/47	59.205	0.000
Surgical duration (min)	128.9 ± 41.6	129.1 ± 44.8	− 0.031	0.975
Perioperative myasthenic crisis, n(%)	32 (10.1%)	17 (12.3%)	0.511	0.475
WHO types of thymoma, n(%)			–	–
A	–	0 (0%)		
AB	–	23 (16.7%)		
B1	–	32 (23.2%)		
B2	–	48 (34.8%)		
B3	–	10 (7.2%)		
Mixed B type	–	24 (17.4%)		
Micronodular thymoma with lymphoid stroma (MNT)	–	1 (0.7%)		
Masaoka-Koga stage, n(%)				
I	–	31 (22.4%)		
II	–	76 (55.1%)		
III	–	27 (19.6%)		
IV	–	4 (2.9%)		

*MG* Myasthenia gravis, *SD* Standard deviation, *VATS* Video-assisted thoracoscopic surgery, *WHO* World Health Organization

### Follow-up data

The median follow-up time following thymectomy was 72 (range, 12–135) months. There were 19 (4.2%) deaths, of whom 7 cases died of MG, 5 cases died of thymoma, 2 cases died of cardiac infarction, 1 case died of lung cancer, 1 case died of gastric cancer, 1 case died of prostatic cancer, 1 case died of pneumonia, and 1 case died of Sjögren’s syndrome. K–M survival analysis demonstrated that thymomatous MG patients had a significantly poorer survival than non-thymomatous MG patients (log-rank, *P* = 0.000).

**Fig. 1:**
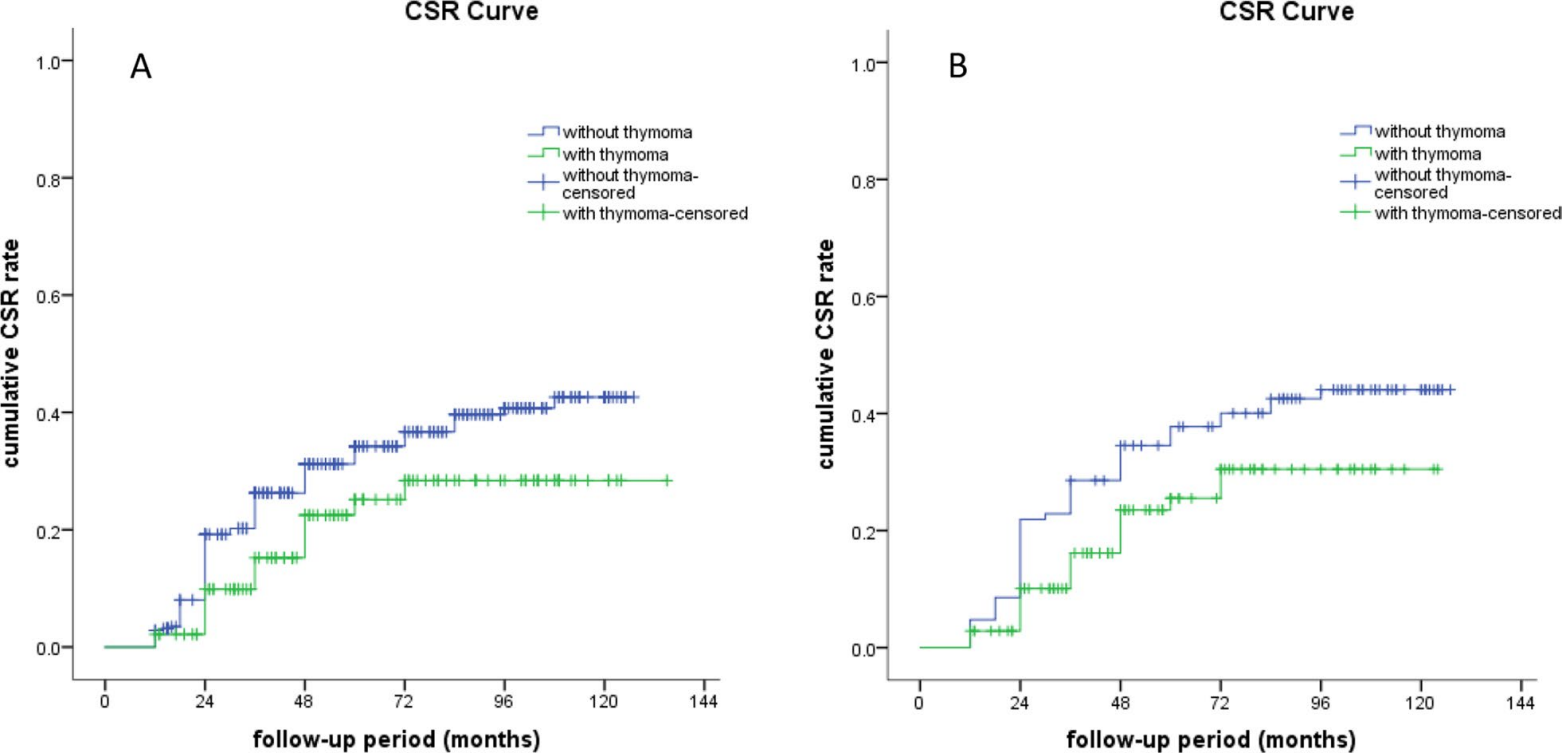
**A** Cumulative CSR rates of thymomatous MG patients and non-thymomatous MG patients before PSM. Thymomatous MG patients had a lower CSR rate compared with non-thymomatous MG patients (log-rank, *P* = 0.019). **B** Cumulative CSR rates of thymomatous MG patients and non-thymomatous MG patients after PSM. Thymomatous MG patients still had a lower CSR rate compared with non-thymomatous MG patients (log-rank, *P* = 0.031)

At the last follow-up, a total of 137 (30.0%) patients attained CSR, 48 (10.5%) patients achieved PR, 149 (32.7%) patients achieved MM, 52 (11.4%) patients had improved, 29 (6.4%) patients were unchanged, 11 (2.4%) patients were worse, 21 (4.6%) patients had exacerbation, and 9 (23.0%) patients died of MG. The effective rate (CSR + PR + MM) was 73.2% (334/456). More non-thymomatous MG patients achieved CSR than thymomatous MG patients (107/318 vs. 30/138, *P* = 0.011). The effective rate was also higher in non-thymomatous MG patients (242/318 vs. 92/138, *P* = 0.037). Considering time to CSR, K–M analysis demonstrated the 3-, 5-, and 8-year cumulative CSR rates were 23.0%, 31.5%, and 37.2%, respectively. Compared with non-thymomatous MG patients, thymomatous MG patients had a significantly lower CSR rate (log-rank, *P* = 0.019)(Fig. 1A). The 3-, 5-, and 8-year cumulative CSR rates were 15.2%, 25.1%, and 28.4% in thymomatous MG patients and 26.3%, 34.2%, and 40.7% in non-thymomatous MG patients, respectively.

### Influential factors for achieving CSR

In univariate Cox analysis of influential factors for achieving CSR, factors including older age at the time of surgery (hazard ratio (HR): 0.974, 95% confidence interval (CI) 0.964–0.985, *P* = 0.000), older onset age of MG (HR:0.511, 95% CI 0.356–0.734, *P* = 0.000), preoperative course of MG > 12 months (HR:0.619, 95% CI 0.421–0.909, *P* = 0.014), presence of thymoma (HR:0.629, 95% CI 0.420–0.943, *P* = 0.025), more preoperative dosage of pyridostigmine bromide used (HR:0.998, 95% CI 0.997–1.000, *P* = 0.030), were associated with a lower possibility of achieving CSR after thymectomy. Influential factors with *P* < 0.1 in univariate analysis were imported into multivariate Cox regression analysis, and the results showed that patients with older age at the time of surgery (HR: 0.971, 95% CI 0.953–0.990, *P* = 0.003), preoperative course of MG > 12 months (HR:0.474, 95% CI 0.317–0.708, *P* = 0.000), and presence of thymoma (HR:0.592, 95% CI 0.389–0.900, *P* = 0.014) had a significantly lower probability of attaining CSR after thymectomy (Table 2).

**Table 2:** Univariate and multivariate Cox analyses of achieving CSR in MG patients

	Univariate analysis			Multivariate analysis		
	HR	95% CI	*P* value	HR	95% CI	*P* value
Age at the time of surgery (years)	0.974	0.964–0.985	0.000	0.971	0.953–0.990	0.003
Sex			0.858			
Male	–	–	–			
Female	1.031	0.738–1.441				
Onset age of MG			0.000			0.895
< 50 years	–	–		–	–	
≥ 50 years	0.511	0.356–0.734		1.042	0.562–1.935	
Preoperative course of MG			0.014			0.000
≤ 12 months	–	–		–	–	
> 12 months	0.619	0.421–0.909		0.474	0.317–0.708	
Thymoma			0.025			0.014
Without thymoma	–	–		–	–	
With thymoma	0.629	0.420–0.943		0.592	0.389–0.900	
Dosage of pyridostigmine bromide(mg/d)	0.998	0.997–1.000	0.030	0.998	0.997–1.000	0.064
Surgical procedure			0.536			
VATS	–	–				
Sternotomy	0.860	0.535–1.385				
Osserman type			0.352			
I + IIa	–	–				
IIb + III + IV	0.853	0.610–1.192				

*MG* Myasthenia gravis, *CSR* Complete stable remission, *HR* Hazard ratio, *CI* Confidence interval, *VATS* Video-assisted thoracoscopic surgery

## Results of PSM

Initially, 105 pairs of patients were matched successfully (Table 3). Clinical features, including age at the time of surgery, gender, onset age of MG, preoperative course of MG, dosage of pyridostigmine bromide, surgical procedure, Osserman class were all adjusted between thymomatous MG patients and non-thymomatous MG patients. K–M analysis demonstrated that thymomatous MG patients still had a poorer OS after matching (log-rank, *P* = 0.048). At the last follow-up, the crude CSR rate and the effective rate (CSR + PR + MM) of thymomatous MG patients were both lower than those of non-thymomatous MG patients (23/105 vs. 44/105, *P* = 0.002; 72/105 vs. 85/105, *P* = 0.039). Considering time to CSR, K–M analysis showed that thymomatous MG patients had a significantly lower CSR rate than non-thymomatous MG patients (log-rank, *P* = 0.048) (Fig. 1B). The 3-, 5-, and 8-year cumulative CSR rates were 16.2%, 25.5%, and 30.5% for thymomatous MG patients and 28.6%, 37.7%, and 44.1% for non-thymomatous MG patients, respectively.

**Table 3:** Comparison of clinical data between thymomatous MG patients and non-thymomatous MG patients after PSM

Variables	Non-thymomatous MG patients	Thymomatous MG patients	T-test or x^2^	*P* value
N	105	105		
Age (years), mean ± SD	50.0 ± 16.8	50.6 ± 13.6	8.884	0.776
Sex (male/female)	53/52	47/58	0.687	0.407
Onset age of MG(< 50 years/ ≥ 50 years)	52/53	48/57	0.305	0.580
Preoperative course of MG (≤ 12 months/ > 12 months)	80/25	85/20	0.707	0.400
Dosage of pyridostigmine bromide(mg/d)	210.8 ± 105.5	213.6 ± 127.9	3.796	0.860
Surgical procedure (VATS/Sternotomy)	85/20	90/15	0.857	0.355
Osserman classification, n (%)		0.020	0.889	
I + IIa	45 (42.9%)	44 (41.9%)		
IIb + III + IV	60 (57.1%)	61 (58.1%)		

*MG* Myasthenia gravis, *SD* Standard deviation, *PSM* Propensity score matching, *VATS* Video-assisted thoracoscopic surgery

## Discussion

Thymoma is the most common type of anterior mediastinal tumors. About 30% of patients with thymoma are accompanied with MG. The clinical features of thymomatous MG patients and non-thymomatous MG patients are quite different [6]. The majority of thymomatous MG patients are diagnosed after 50 years old, and are without male or female sex predominance. Symptoms in thymomatous MG patients are typically more severe, accompanying by a higher frequency of generalized disease with bulbar and respiratory symptoms [16]. Similar clinical features were observed in the present study. The age at the time of surgery was older, and the preoperative course of MG was shorter in thymomatous MG patients. Thymomatous MG patients were more likely to develop severe symptoms (Osserman class IIb + III + IV), and a higher proportion underwent thymectomy by sternotomy.

Previous studies demonstrated that the existence of thymoma complicates the treatment of MG, and may have a negative influence on neurological outcome of MG patients [7–10]. Thymomatous MG patients were prone to have severe clinical symptoms. And based on former studies, it seems that the more severe the disease is, the worse the outcome. Furthermore, many patients with thymomas were introduced to receive postoperative chemotherapy or radiotherapy. Those therapies can lead to exacerbation of MG symptoms, which may affect the outcome of MG. In the present study, a large MG cohort with a long-time follow-up was investigated. At the last follow-up, the crude CSR rate of thymomatous MG patients (21.3%) was lower than that of non-thymomatous MG patients (33.3%). Furthermore, considering time to CSR, K–M analysis showed that the cumulative CSR rate was also significantly lower in thymomatous MG patients. As mentioned earlier, several clinical features were different between the two groups, which might confound the results of analyzing how thymoma could affect the surgical effects on MG. To eliminate selection bias, PSM was used to obtain new paired patients with adjusted clinical variables. Comparison between the two matched groups revealed that, the crude CSR rate, cumulative CSR rate and the effective rate (CSR + PR + MM) were all lower in thymomatous MG patients than those in non-thymomatous MG patients. Considering CSR as the primary endpoint, Cox analysis was performed and demonstrated that thymoma was a negative predictive factor for CSR (HR: 0.592, 95% CI 0.389–0.900, *P* = 0.014). Therefore, it could be concluded that thymoma negatively affected the neurological effect of MG after thymectomy. However, some studies could not draw similar conclusions [11–13], which showed that thymoma did not significantly affect the neurological outcome of MG. The different characteristics of MG cohorts included in different studies may contribute to various results. Besides, medications for MG have improved rapidly these years, which can lead to overall improvement of MG prognosis. In fact, the pathophysiological mechanism of thymoma affecting MG prognosis is not well understood. There are still many issues that need to be studied in the relationship between MG and thymoma.

Other factors may potentially affect the clinical outcome of thymectomy. Some studies pointed out that a younger age at the time of surgery and a shorter time interval between diagnosis and thymectomy were favorable variables for achieving CSR after thymectomy [17–20], and similar results were obtained in the present study by performing univariate and multivariate Cox analyses. Some scholars demonstrated that female patients and patients with mild symptoms or those receiving a lower dose of pyridostigmine bromide may have better outcome after thymectomy [7, 19–21]. However, multivariate Cox analysis did not confirm similar results in the present study. The discrepancies among different studies may be partly attributed to small sample size and the differences in study population.

This was the first research using PSM to evaluate how thymoma could affect the neurological outcome of MG after thymectomy, with a long-time follow-up. Several limitations of the present study should be pointed out. First, this was a retrospective single-center study, and selection bias was unavoidable. Second, results of testing of auto-antibodies were lacking for many patients in this study. No analysis of auto-antibodies was performed. Third, medications for MG after thymectomy were not standard, and several drugs may be prescribed at the same time or successively during a long-time follow-up period after thymectomy, which made it impossible to analyze this data. Prospective studies with standard therapeutic strategies after thymectomy are required to evaluate the influential factors more precisely.

## Conclusions

The concurrent thymoma had a negative effect on the neurological outcome of MG after thymectomy. MG patients with old age and a preoperative course of longer than one year had a lower probability of achieving CSR.

## Data Availability

All data generated or analysed during this study are included in this published article. The datasets used during the current study are available from the corresponding author on reasonable request.
